# The Cytosolic Domain of Pex22p Stimulates the Pex4p-Dependent Ubiquitination of the PTS1-Receptor

**DOI:** 10.1371/journal.pone.0105894

**Published:** 2014-08-27

**Authors:** Fouzi El Magraoui, Andreas Schrötter, Rebecca Brinkmeier, Lena Kunst, Thomas Mastalski, Thorsten Müller, Katrin Marcus, Helmut E. Meyer, Wolfgang Girzalsky, Ralf Erdmann, Harald W. Platta

**Affiliations:** 1 Biochemie Intrazellulärer Transportprozesse, Ruhr-Universität Bochum, Bochum, Germany; 2 Systembiochemie, Ruhr-Universität Bochum, Bochum, Germany; 3 Medizinische Proteomik/Bioanalytik, AG Neuro Proteomics, Medizinisches Proteom-Center, Bochum, Germany; 4 Funktionelle Proteomik, Medizinisches Proteom-Center, Bochum, Germany; 5 Biomedizinische Forschung, Leibniz-Institut für Analytische Wissenschaften - ISAS -e.V., Dortmund, Germany; Centre National de la Recherche Scientifique, Aix-Marseille Université, France

## Abstract

Peroxisomal biogenesis is an ubiquitin-dependent process because the receptors required for the import of peroxisomal matrix proteins are controlled via their ubiquitination status. A key step is the monoubiquitination of the import receptor Pex5p by the ubiquitin-conjugating enzyme (E2) Pex4p. This monoubiquitination is supposed to take place after Pex5p has released the cargo into the peroxisomal matrix and primes Pex5p for the extraction from the membrane by the mechano-enzymes Pex1p/Pex6p. These two AAA-type ATPases export Pex5p back to the cytosol for further rounds of matrix protein import. Recently, it has been reported that the soluble Pex4p requires the interaction to its peroxisomal membrane-anchor Pex22p to display full activity. Here we demonstrate that the soluble C-terminal domain of Pex22p harbours its biological activity and that this activity is independent from its function as membrane-anchor of Pex4p. We show that Pex4p can be functionally fused to the trans-membrane segment of the membrane protein Pex3p, which is not directly involved in Pex5p-ubiquitination and matrix protein import. However, this Pex3(N)-Pex4p chimera can only complement the double-deletion strain *pex4*Δ/*pex2*2Δ and ensure optimal Pex5p-ubiquitination when the C-terminal part of Pex22p is additionally expressed in the cell. Thus, while the membrane-bound portion Pex22(N)p is not required when Pex4p is fused to Pex3(N)p, the soluble Pex22(C)p is essential for peroxisomal biogenesis and efficient monoubiquitination of the import receptor Pex5p by the E3-ligase Pex12p *in vivo* and *in vitro*. The results merge into a picture of an ubiquitin-conjugating complex at the peroxisomal membrane consisting of three domains: the ubiquitin-conjugating domain (Pex4p), a membrane-anchor domain (Pex22(N)p) and an enhancing domain (Pex22(C)p), with the membrane-anchor domain being mutually exchangeable, while the Ubc- and enhancer-domains are essential.

## Introduction

Ubiquitination is one of the most prominent posttranslational modifications within the cell. It regulates the targeting and in many cases also the stability of proteins. The attachment of this modification requires a three-step enzyme cascade. First, the ubiquitin-activating enzyme (E1) forms an AMP-ubiquitin intermediate before it binds the activated ubiquitin moiety via a thioester-bond. Thereafter, ubiquitin is bound to the active-site cysteine of an ubiquitin-conjugating enzyme (E2). The final transfer of the ubiquitin moiety to the substrate usually requires the activity of an ubiquitin-protein ligase (E3). The E3 enzyme binds both the Ub-charged E2 enzyme as well as the target protein and either enables a direct transfer (RING-type E3) or requires a thioester-linked Ub-E3 intermediate (HECT-type and RBR-type E3s) in order to finally attach the ubiquitin moiety to the target amino acid of the substrate [1]–[3]. In recent years examples of additional, non-canonical ubiquitination factors have been described, comprising *e.g.* additional E3 enzymes (E4) like Ufd2p [4] or E2-activators like Cue1p [5], [6].

Ubiquitination plays a central role in the biogenesis of peroxisomes. In fact, the first E2 enzyme required for the formation of an organelle, namely Pex4p (Pas2p/Ubc10p), has been found to be functionally linked to peroxisomal matrix protein import [7].

Peroxisomes are present in almost all eukaryotic cells and their dysfunction in humans is associated with a spectrum of severe peroxisomal disorders [8]–[10]. More than 50 different enzymes have been described to be compartmentalized in the peroxisomal matrix [11] which link this organelle to diverse biochemical reaction pathways and physiological functions. The most prominent task is the breakdown of fatty acids and the detoxification of hydrogen-peroxide [12], [13].

The operativeness of this organelle is governed by dynamically operating import machineries for peroxisomal membrane and matrix proteins. All peroxisomal proteins are synthesized on free ribosomes in the cytosol and usually harbour a peroxisomal targeting sequence (PTS). Peroxisomal membrane proteins contain the mPTS, which comprises the binding site to the membrane protein-receptor/chaperone Pex19p as well as a membrane-association region [14], [15]. Matrix proteins harbor either a PTS1 or a PTS2 sequence, which is recognized by the soluble PTS1-receptor Pex5p or the PTS2-receptor Pex7p, respectively [12], [13], [16]. Most peroxisomal matrix proteins are imported by the PTS1-receptor Pex5p. This cycling receptor binds its cargo proteins in the cytosol and ferries them to the peroxisome. The cargo is thought to be transported over the membrane via a transiently opened import pore [17] and finally released into the peroxisomal matrix. The PTS1-receptor is exported back to the cytosol in order to facilitate further rounds of matrix protein import [18]. This dislocation step is accomplished by the concerted action of peroxisomal membrane subcomplexes, collectively referred to as the peroxisomal exportomer [19]. This molecular machinery comprises enzymes required for the ubiquitination as well as the ATP-dependent extraction of the receptor from the membrane. A prerequisite for the export step is the monoubiquitination of the PTS1-import receptor Pex5p on a conserved cysteine [20]–[23]. Also the PTS2-receptor module has been shown to be regulated by monoubiquitination [24]–[26]. In yeast, the monoubiquitination of the PTS1-receptor Pex5p is catalyzed by the E2 enzyme Pex4p [22], [23]. Pex4p cooperates with the peroxisomal RING-type E3 complex, from which Pex12p has been demonstrated to directly ubiquitinate Pex5p [27]. The monoubiquitinated Pex5p is recognized by the AAA-type ATPases Pex1p and Pex6p and dislocated to the cytosol [20]–[22], [28], [29]. In case this export reaction is affected, like *e.g.* in a *pex1*Δ or *pex4*Δ strain, Pex5p becomes a substrate of a quality control pathway, resulting in its polyubiquitination on lysine residues and subsequent degradation by the 26S proteasome [22], [23], [30], [31]. Interestingly, the deletion of the membrane protein Pex22p displays a similar phenotype [30], [31], most likely due to its known role as anchor protein of Pex4p [32], [33]. Moreover, it has been shown recently that Pex4p requires the interaction with Pex22p to display full activity [34], [35].

Here we demonstrate that the soluble carboxyl (C)-terminal domain of Pex22p harbours its biological activity. We find that Pex4p can even be genetically fused to the membrane anchor portion of the membrane protein Pex3p and is still capable to complement the *pex4*Δ deletion strain. Interestingly, the *pex4*Δ*pex22*Δ double deletion strain can be complemented by these chimeric fusion constructs only in case the soluble C-terminal part of Pex22p is additionally expressed in the cell. Thus, while the membrane anchoring, amino (N)-terminal portion Pex22(N)p is not required when Pex4p is fused to Pex3(N)p, the soluble Pex22(C)p is essential for peroxisomal biogenesis and efficient monoubiquitination of the PTS1-import receptor Pex5p.

## Materials and Methods

### Yeast strains and culture conditions

The *Saccharomyces cerevisiae* strain UTL-7A (MATa, ura3–52, trp1, leu2–3/112) was used as wild-type strain for the generation of several isogenic deletion strains by the ‘short flanking homology’ method as described [36]. The resulting deletion strains were *pex4*Δ [7], *pex5*Δ [37], *pex22*Δ and *pex4*Δ/*pex22*Δ [31]. Yeast complete (YPD) and minimal media (SD) as well as oleic acid medium (YNO) have been described previously [38].

### Complementation activity

The functionality of peroxisomes was monitored by measuring the OD600 of the cells grown in YNO. Cells were first precultured for 16 h in 25 ml SD medium and transferred to 500 ml YNO with a starting OD600 of 0.1. OD600 was measured after 24 hrs and data were corrected for the measured value of the negative control. The corrected data are displayed in % complementation activity compared to wild-type cells.

### Plasmids, cloning strategies and recombinant proteins

The generation of the constructs coding for GST-Pex12p(aa293–399) [39], GST-Pex4p [22], His-Pex5p [40], DsRed-PTS1 as well as DsRed-Ant1p [41] have been described previously. In order to generate the Pex3(1–45)-Pex4p-GFP construct, Pex4p was amplified from genomic DNA using the primer pair RE2417/RE2416. The obtained product was cloned with *Bam*HI/*Sal*I into the pAH37 vector (pUG35-Pex3(1–45)-GFP) [41]. The Pex3(1–45)-Pex4p construct was generated by the same procedure, with the only difference that Pex4p was amplified with its stop codon using the primer pair RE2418/RE2416. In order to generate the Pex22(1–35)-Pex4p-GFP construct, Pex4p was amplified from genomic DNA using the primer pair RE2417/RE2416. The obtained product was cloned *Bam*HI/*Sal*I into the pAH44 vector (pUG35-Pex22(1–35)-GFP) [41]. The Pex3(1–45)-Pex4p construct was generated by the same procedure, with the only difference that Pex4p was amplified with its stop codon using the primer pair RE2418/RE2416. The Pex22p(36–180) construct was generated via amplification of the Pex22p-fragment from genomic DNA using the primer pair RE1222/RE3441. The isolated fragment was cloned via *Bam*HI/*Xba*I restriction in a pRS415 vector, which contains a *Sac*I/*Spe*I-inserted *MET25*-promotor.

In order to generate the His_6_-Pex22(36–180)p construct, the fragment coding for the soluble domain of Pex22p was amplified from genomic DNA using the primers KU1164 and KU1165. The PCR fragment was cloned via *Nco*I and *Bam*HI into pET9d (Novagen).

Primer (Eurogentec) used in this study:

- KU1164 TAACCATGGAAAAGGTGACAAGTGCAAAG
- KU1165 TAAGGATCCTTAATTGCATAAAGTGTCAAT
- RE1222 AAGTCGACATCTAGAATGACAAGTGCAAAGGAAGATA
- RE2416 AAGATCCATGCCAAACTTCTGGATTCTT
- RE2417 CCGTCGACATGGTTGTTGATCCGCTC
- RE2418 AAGGTACCTCAATGGTTGTTGATCCGCTC
- RE3441 AAGGATCC TTAATTGCATAAAGTGTCAATCAGC

### Recombinant proteins

Genes coding for recombinant proteins were expressed in *E. coli* BL21(DE3). The procedures for the preparation of GST-Pex12p(aa293–399) and GST-Pex4p [22] as well as for His-Pex22p(aa36–180) and His-Pex5p [27] have been described previously. Recombinant yeast E1 and ubiquitin were purchased from Sigma (München, Germany).

### *In vitro* ubiquitination

Autoubiquitination assays of Pex4p were carried out as described previously [22] with the difference that 0.8 µg His-Pex22(C)p were added to the indicated reactions. *In vitro* ubiquitination of recombinant His-Pex5p contained 0.5 µg of His-Pex5p, 0.5 µg of GST-RING fusion proteins, 0.1 µg of yeast E1, 0.8 µg E2-enzyme as indicated, 5 µg of ubiquitin-species as indicated and 10 µl buffer containing 2 mM ATP, 50 mM Tris–HCl pH 7.5, 2 mM MgCl_2_, 2 µM ZnCl_2_ and 0.1 mM dithiothreitol. After incubated at 30°C for 90 min with gentle shaking, the reaction was stopped by addition of SDS-sample buffer and heating at 95°C for 5 min.

### *In vivo* ubiquitination

Preparation of polyubiquitinated Pex5p from oleate-induced yeast was based on a trichloroacetic acid precipitation and has been described previously [31]. Preparation of monoubiquitinated myc-Pex5p was performed by immunoprecipitation with myc-antibodies and Dynabeads anti-mouse-IgGs (Life Technologies) in the presence of 20 mM NEM.

### Microscopy

Analysis of live cells for dsRed and GFP fluorescence was performed with a Zeiss Axioplan microscope and AxioVision 4.1 software (Zeiss, Jena, Germany). Before inspection, cells were grown for 2 days on solid minimal medium containing oleic acid as a sole carbon source [41].

### Mass Spectrometry

The sample of the complex isolation was deluted by LDS-Puffer (“NuPAGE LDS Sample Buffer 4x” von Invitrogen/Life Technologies, Carlsbad, USA), heated for 5 minutes on 95°C and separated by SDS-PAGE with a 4–12% NuPAGE Bis-Tris Gel (Invitrogen). After 18 minutes of separation at 50 V, the gel was stained with Imperial Protein Stain (Thermo Scientific) and the bands were cut, destained and in-gel digested with trypsin (Promega). Peptide extraction, sample preparation and liquid chromatography-mass spectrometry (LC-MS/MS) was performed as described [42].

### Immunodetection

Polyclonal rabbit antibodies were raised against Pex5p [43], Pex22p (this study) and ubiquitin (Sigma, München, Germany). Monoclonal mouse antibodies were used for detection of GST (Sigma, München, Germany). Immuno-reactive complexes were visualized using the IRDye 800CW goat anti-rabbit IgG or IRDye 680 goat anti-mouse secondary antibody (Li-COR Bioscience, Bad Homburg, Germany) followed by detection using the “Infrarot Imaging System” (Li-COR Bioscience, Bad Homburg, Germany).

## Results

### The chimeric Pex3(N)-Pex4p fusion protein is targeted to peroxisomes and attracts the soluble Pex22(C)p

The soluble ubiquitin-conjugating enzyme Pex4p associates with peroxisomes via its interaction to the C-terminal part of the peroxisomal membrane protein Pex22p [32]. The binding to Pex22p enhances the activity of Pex4p [34], [35]. We wanted to analyze the functional impact of this interaction on the assembly of the Pex4p/Pex22p complex as well as on the Pex12p-dependent ubiquitination of the PTS1-receptor Pex5p.

**Figure 1:**
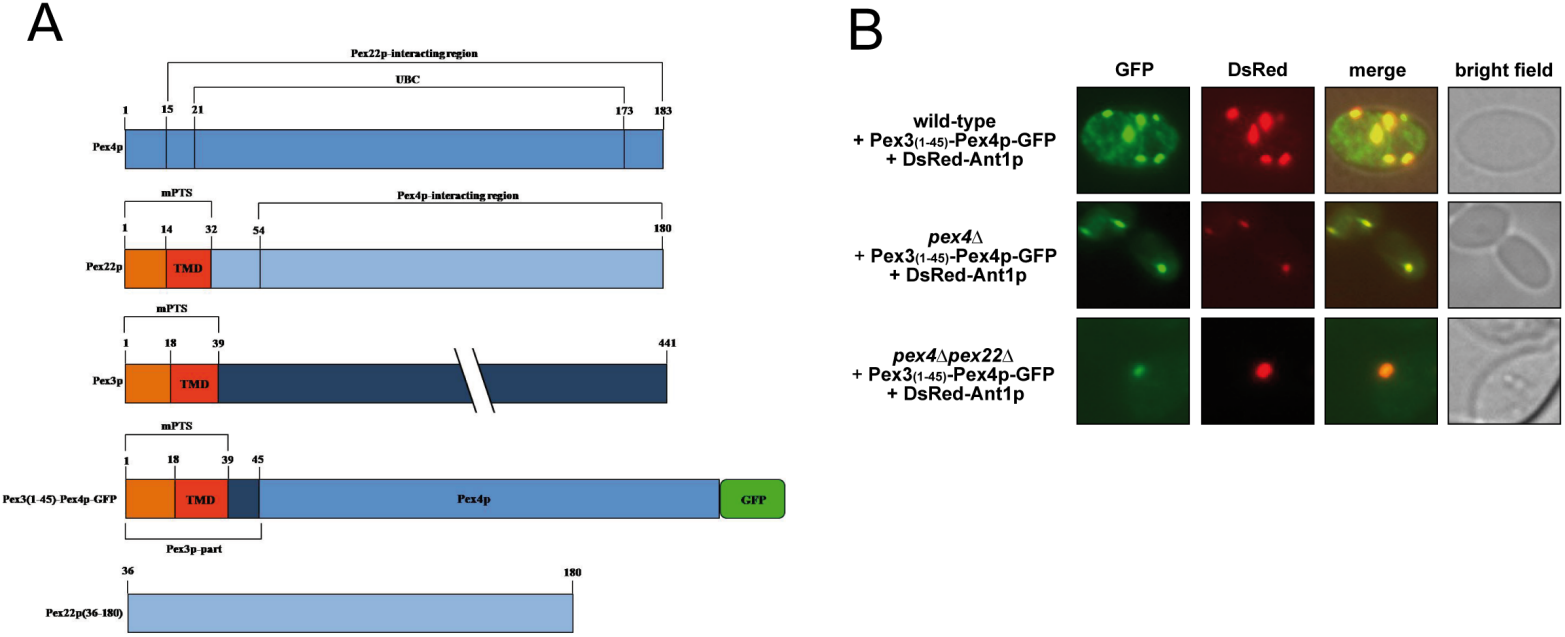
The Pex3(1–45)-Pex4p fusion protein binds the soluble Pex22(C)p-fragment at peroxisomes. (**a**) Schematic topological representations of full-length Pex4p and the applied chimeric and truncated versions of Pex4p, Pex3p and Pex22p. The luminal domains are shown in *orange,* the trans-membrane domains (TMD) in *red*, the cytosolic parts in *blue* and GFP (green fluorescent protein) in *green*. The membrane protein targeting signal (mPTS), the ubiquitin-conjugating domain (UBC) as well as numbers of important amino acid positions are denoted. Note: While the domains of Pex22p, Pex4p and Pex3p are depicted at the same scale, the size of GFP (green fluorescent protein) is reduced in this model. (**b**) Peroxisomal targeting of a chimeric Pex3(1–45)-Pex4p. Intracellular localization of chimeric Pex4p in wild-type and indicated *pex*-mutant cells is monitored by fluorescence microscopy. Pex4p is usually anchored to peroxisomes via binding to the C-terminal part of the peroxisomal membrane protein Pex22p. Colocalization of the GFP-tagged chimeric Pex3p(1–45)-Pex4p with the DsRed-tagged peroxisomal membrane marker Ant1p indicates that it localized to peroxisomes in all strains.

For this purpose, we investigated whether the function of Pex22p as Pex4p-anchor can be uncoupled from its function as Pex4p-enhancer. To this end, we generated a chimeric protein, consisting of Pex4p and the N-terminal amino acids 1–45 of Pex3p (Pex3p(N)-Pex4p) and GFP for detection (Fig. 1a). In *S. cerevisiae*, Pex3p takes part in the process of membrane protein insertion, *de novo* formation of peroxisomes, pexophagy and inheritance, but is not directly involved in matrix protein import [44]–[48]. The N-terminal 45 amino acids of Pex3p contain its mPTS and membrane anchor sequence [41], [49] (Fig. 1a). Thus fusion of this region to Pex4p-GFP should direct the protein to peroxisomes and anchor the fusion protein to the peroxisomal membrane. Accordingly, we analyzed the peroxisomal targeting of the Pex3(N)-Pex4p-GFP fusion protein to peroxisomes in wild-type, *pex4*Δ and *pex4*Δ*pex22*Δ strains (Fig. 1b). For this, the GFP-fused Pex4p-chimera was assayed for co-localization with the DsRed-tagged peroxisomal adenine nucleotide transporter Ant1p. The fluorescence microscopic analysis revealed that Pex3(N)-Pex4p-GFP was targeted to peroxisomes in all tested strains, which indicated that the Pex3(N)-anchored Pex4p-GFP form is targeted the peroxisomal membrane in cells that completely lack Pex22p (Fig. 1b). Thus, under these circumstances Pex22p is dispensable for peroxisomal targeting of Pex4p, which now is membrane anchored by the Pex3p(1–45). Furthermore, we tested, if the detached soluble carboxyl-terminal part of Pex22(aa36–180)p ( = Pex22(C)p) can bind to the peroxisomal Pex4p-chimera when it is additionally expressed within the cell. The Pex22(C)p-fragment mainly contains the binding region required for the interaction with Pex4p [34], but no membrane domain (Fig. 1a). To test for the binding capabilities, we isolated Pex3(N)-Pex4p-GFP via immunoprecipitation with GFP-antibodies from *pex4*Δ*pex22*Δ cells that also expressed Pex22(C)p and analyzed the isolated complex by mass-spectrometry. Peptides derived from Pex22(C)p were detected in samples derived from cells that contained Pex3(N)-Pex4p-GFP, while no Pex22(C)p was detected in samples from strains that had not been transformed with the Pex4-GFP-chimera (data not shown). Therefore, we can conclude that Pex22(C)p binds via Pex3(N)-Pex4p-GFP to peroxisomes.

Taken together these data demonstrate that Pex4p can target to peroxisomes independently of Pex22p when it is fused to the membrane-anchor Pex3(N)p and that Pex22(C)p can associate with this assembly.

### The function of Pex22(N)p is dispensable for matrix protein import, while Pex22(C)p is essential for biological activity

**Figure 2:**
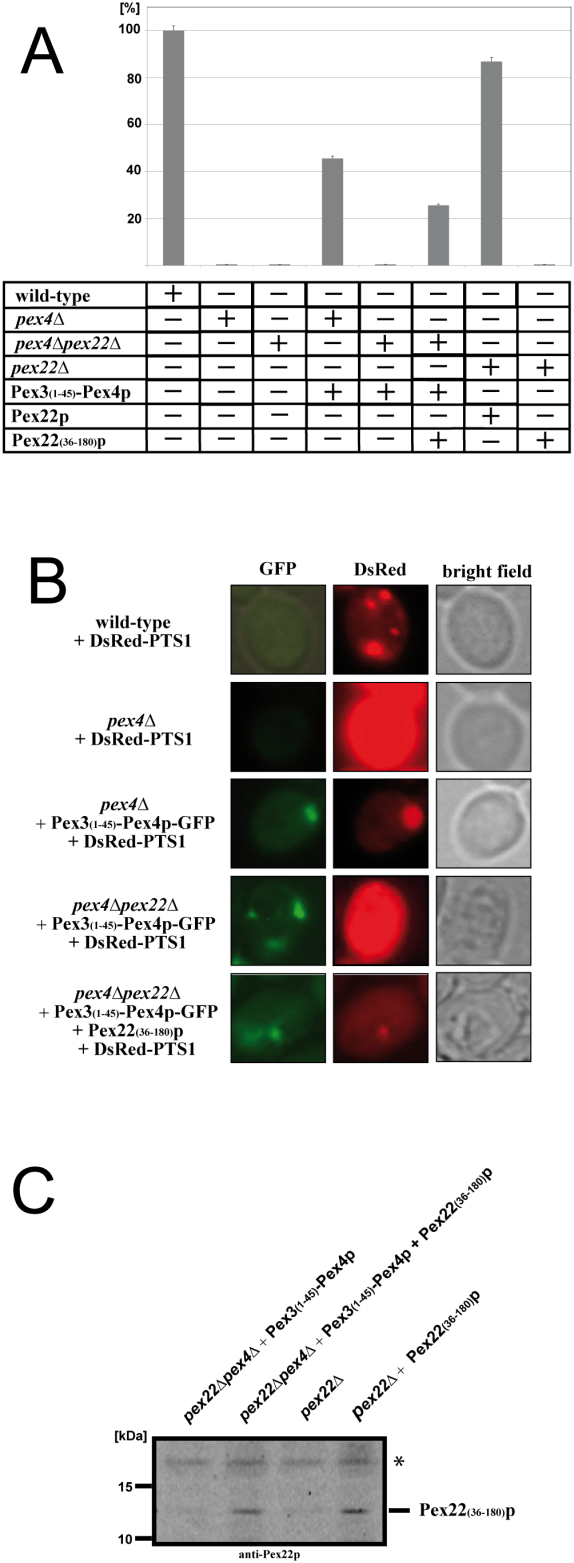
The function of Pex22(C)p is essential for peroxisome biogenesis, while Pex22(N)p is dispensable. (**a**) Peroxisomal function was tested by analyzing the capability of the plasmid-encoded constructs to complement the mutant growth phenotype of corresponding deletion strains. To this end, the Pex3(N)-Pex4p, Pex22(C)p and/or Pex22p were expressed in the *pex4*Δ*pex22*Δ, *pex4*Δ *or pex22*Δ deletion strains, as indicated. The optical density at 600 nm (OD600) of cells grown in oleate medium was monitored (n = 3 experiments) and results are presented in relation to growth of the wild-type, which is set as 100% (+/−standard error of the mean). The corresponding OD600 at the end of the growth period is depicted. While Pex3(N)-Pex4p can complement the growth defect of the *pex4*Δ strain, it is only capable to regain functionality in the *pex4*Δ*pex22*Δ background, when Pex22(C)p is present. (**b**) Peroxisomal function was tested by analyzing the capability of the plasmid-encoded constructs to complement the peroxisomal protein import defect of corresponding deletion strains. Plasmid-encoded DsRed-PTS1 served as marker for peroxisomal protein import. Transformed cells were grown on oleic acid plates for two days and examined by fluorescence microscopy. The wild-type cells as well as the *pex4*Δ strain carrying the Pex3(N)-Pex4p plasmid displayed a punctate pattern, indicating that DsRed-PTS1 cargo is imported into peroxisomes and which is the typical appearance of cells with intact PTS1-dependent matrix protein import. The non-transformed *pex4*Δ strain as well as the *pex4*Δ*pex22*Δ strain with Pex3(N)-Pex4p displayed a cytosolic staining of the DsRed-signal, demonstrating that the marker is mislocalized to the cytosol due to a block of import. The *pex4*Δ*pex22*Δ strain expressing both Pex3(N)-Pex4p and Pex22(C)p displayed a heterogenous phenotype of a partial cytosolic mislocalization but also detectable import of DsRed-PTS1 into peroxisomes. (**c**) The lysates of oleate-induced cells were analyzed for the level of Pex22(C)p. The detected level of Pex22(C)p was the same in both the strain with partial functional complementation (*pex4*Δ*pex22*Δ+Pex3(N)-Pex4p+Pex22(C)p) and the strain without functional complementation (*pex22*Δ+Pex22(C)p). The asterisk denotes a cross-reaction signal.

In order to determine the functionality of the Pex4p-chimera complex in biogenesis of peroxisomes, we tested the complementation activity of Pex3(N)-Pex4p in combination with Pex22(C)p in *pex4*Δ and *pex4*Δ*pex22*Δ strains in comparison to wild-type cells (Fig. 2a). The cells were incubated in growth media that contained oleate as sole carbon source. Under these conditions peroxisomes become essential for the viability of the cells because they are the only site where fatty acids can be degraded via beta-oxidation [50]. Growth of the wild-type is set as 100% (Fig. 2a). As *pex4*Δ and *pex4*Δ*pex22*Δ strains are affected in peroxisome biogenesis, they do not grow on oleate-medium. The *pex4*Δ cells containing Pex3(N)-Pex4p can grow on oleate-medium, indicating that the chimeric protein can functionally account for the absence of the endogenous Pex4p. However, even in the presence of the chimeric protein, peroxisomal biogenesis is still hampered when PEX22 is deleted in addition to PEX4. Because the cellular level of the Pex4p-chimera in *pex4*Δ and *pex4*Δ*pex22*Δ cells remains the same (data not shown), the observed biogenesis defect is not the result of an instability of the Pex4p-chimera but is more likely caused by a function of Pex22p that is independent of its role as membrane anchor of Pex4p. We assumed that the C-terminal region of Pex22p might account for this function. Accordingly, we analyzed the effect of Pex22(aa36–180)p, in the following named Pex22(C)p. This truncated Pex22p still contains the C-terminal domain but lacks the N-terminal regions for peroxisomal targeting and membrane insertion. When this construct is expressed in *pex4*Δ*pex22*Δ cells together with the chimeric Pex3(N)-Pex4p, the cells regained the ability to grow on oleate medium (Fig. 2a). This restoration does not reach the wild-type level, which is likely caused by the lower efficiency of the peroxisomal targeting of Pex22(C)p. In a control experiment, we verified that the cellular level of overexpressed Pex22(C)p is the same in all tested strains (Fig. 2c). Therefore, complementation can only be achieved when the peroxisomal E2-complex can form (*e.g.* in *pex4*Δ*pex22*Δ+Pex3(N)−Pex4p+Pex22(C)p) and is not possible when Pex4p is mislocalized, even if Pex22(C)p is present (*e.g.* in *pex22*Δ+Pex22(C)p).

Importantly, these data demonstrate that membrane-anchored Pex4p can perform its function during peroxisomal biogenesis independently of full-length Pex22p as long as it can associate with Pex22(C)p, even when its membrane-spanning part Pex22(N)p is completely absent.

In order to analyze whether the observed complementation of the growth phenotype correlates with restoration of peroxisome biogenesis and peroxisomal protein import, we monitored transformed wild-type and mutant cells for the intracellular localization of the model cargo DsRed-PTS1 by direct fluorescence microscopy (Fig. 2b). In wild-type cells, the punctate pattern indicates a complete import of the PTS1-containing fluorophore into peroxisomes (Fig. 2b), while the deletion of peroxin genes prevents the import and results in a cytosolic mislocalization as seen here for non-complemented *pex4*Δ cells. The punctate patter observed for DsRed-PTS1 in the *pex4*Δ strain transformed with Pex3(N)-Pex4p-GFP demonstrate that these cells display a functional matrix protein import and thus restoration of the peroxisome biogenesis defect (Fig. 2b). However, Pex3(N)-Pex4p-GFP was not able to rescue the DsRed-PTS1 import in the *pex4*Δ*pex22*Δ background. The additional presence of the Pex22(C)p construct displayed a heterogenous phenotype. Even though a significant amount of DsRed-PTS1 was still mislocalized to the cytosol, the peroxisomes were nevertheless found to be import competent (Fig. 2b). These data correlate with our finding that the complementation of Pex3(N)-Pex4p combined with the Pex22(C)p fragment in the *pex4*Δ*pex22*Δ background is only partial (Fig. 2a).

To conclude, the data corroborate the complementation assays by growth analysis in that they demonstrate that the chimeric Pex3(N)-Pex4p is functional in the presence of Pex22p and that under these conditions the Pex22(C)p fragment can account for the requirement for Pex22p. These data clearly show that Pex22p is not merely a docking protein for the ubiquitin conjugating Pex4p but that its C-terminal domain maintains an important function in peroxisome biogenesis.

### Pex22(C)p stimulates the autoubiquitination of Pex4p as well as the Pex12p-dependent ubiquitination of the PTS1-receptor *in vitro*

**Figure 3:**
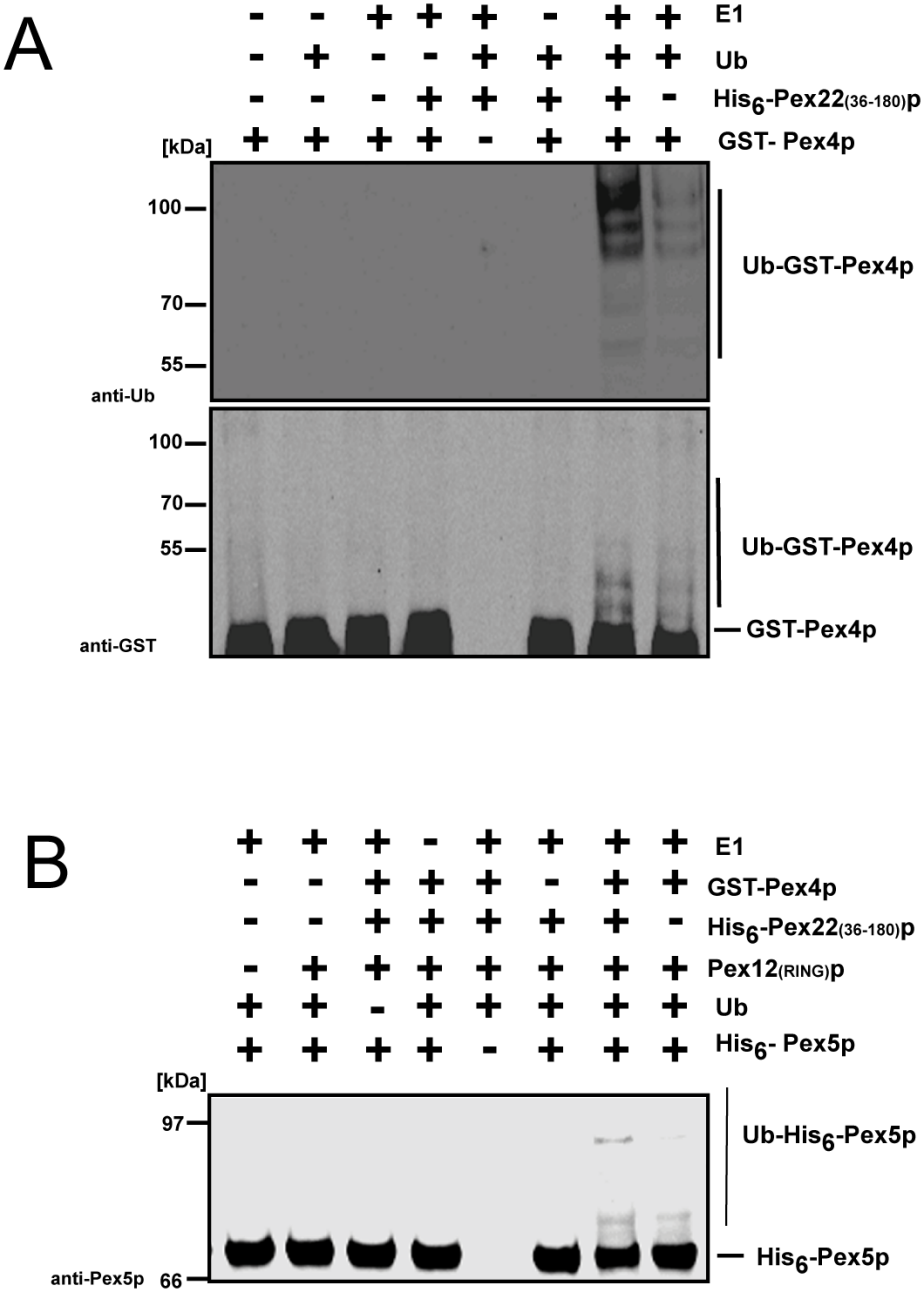
Pex22(C)p enhances autoubiquitination of Pex4p and Pex4p/Pex12p-catalyzed ubiquitination of the PTS1-receptor *in vitro*. (**a**) Autoubiquitination of GST-Pex4p. The effect of Pex22(C)p on the autoubiquitination of GST-Pex4p was examined *in vitro* using heterologously expressed and purified proteins as indicated. Autoubiquitination of GST-Pex4p depends on the presence of E1 and ubiquitin (Ub). As shown by immunoblot analysis with antibodies against GST and Ub, the autoubiquitination of GST-Pex4p is significantly enhanced in the presence of His-Pex22(C)p. (**b**) Ubiquitination of the PTS1-receptor Pex5p. Recombinant proteins were used for *in vitro* ubiquitination of Pex5p, which depends on the presence of E1, GST-Pex4p, Ub as well as the RING-domain of the E3 enzyme Pex12p and was significantly enhanced in presence of His-Pex22(C)p.

As Pex22p interacts with Pex4p, we investigated whether the detected function of Pex22(C)p in matrix protein import is connected to Pex4p-mediated ubiquitination events. First, we tested the effect of Pex22(C)p on the *in vitro* autoubiquitination of Pex4p (Fig. 3a). For this purpose, we purified heterologously expressed GST-tagged Pex4p, His-tagged Pex22(C)p as well as His-tagged Pex5p and the RING-domain of Pex12p from *E. coli* and analyzed them in an *in vitro* ubiquitination assay in different combinations. The generated protein samples were subjected to immunoblot analysis with GST- and ubiquitin-antibodies. The data set shows that the *in vitro* autoubiquitination reaction of GST-Pex4p depends on the presence of E1 and ubiquitin (Fig. 3a). Furthermore, His-Pex22(C)p does not display any ubiquitination activity on its own, but its addition to the reaction sample containing GST-Pex4p, E1 and ubiquitin significantly enhances the autoubiquitination of the peroxisomal E2 enzyme. Thus, even though His-Pex22(C)p does not display intrinsic ubiquitination activity, it is capable to stimulate the autoubiquitination activity of GST-Pex4p (Fig. 3a).

Next we wanted to analyze whether Pex22(C)p also has an effect on the ubiquitination of the PTS1-receptor Pex5p, which is a well-known target protein for Pex4p-dependent ubiquitination [22], [23]. The *in vitro* ubiquitination assays demonstrated that the ubiquitination of His-Pex5p depends on the presence of E1, GST-Pex4p, ubiquitin as well as the RING-domain of the E3-enzyme Pex12p [27] (Fig. 3b). Under these conditions a small portion of Pex5p is ubiquitinated (Fig. 3b, last lane). Moreover, the data reveal that His-Pex22(C)p does not ubiquitinate His-Pex5p itself, as no modification is visible when GST-Pex4p is omitted from the His-Pex22(C)p-containing sample (Fig. 3b, lane 6). However, in the presence of GST-Pex4p and His-Pex22(C)p, a clear increase in the intensity of the band representing ubiquitinated Pex5p is observed in the presence of Pex4p and His-Pex22(C)p (Fig. 3b, lane 7). The result shows that His-Pex22(C)p stimulates the GST-Pex4p-mediated *in vitro* ubiquitination of Pex5p (Fig. 3b). We conclude that Pex22(C)p can not only stimulate the autoubiquitination of Pex4p, but also that this enhancing effect can be directly transferred to the Pex12p-dependent ubiquitination of the PTS1-receptor Pex5p.

### Pex22(C)p reduces polyubiquitination and stimulates monoubiquitination of Pex5p *in vivo*

**Figure 4:**
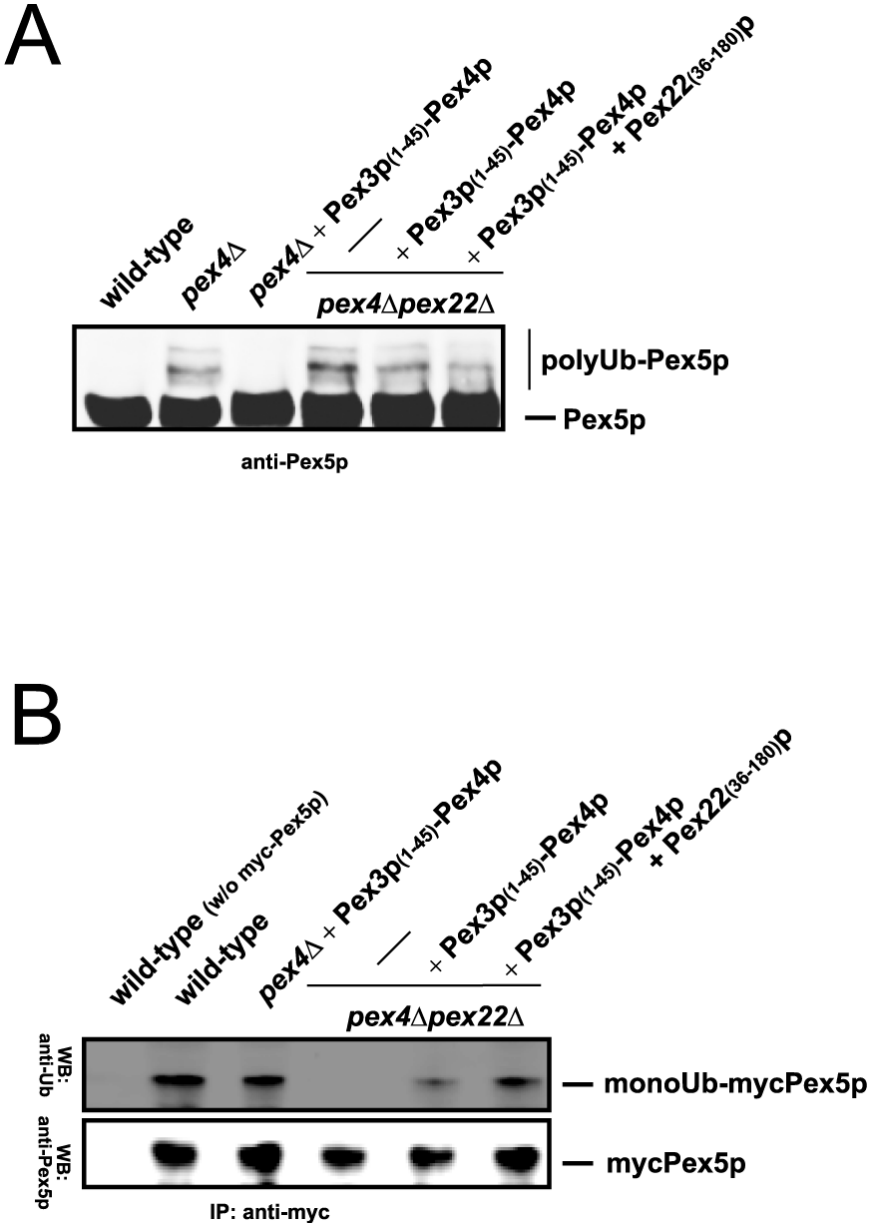
Pex22(C)p reduces polyubiquitination and stimulates monoubiquitination of the PTS1-receptor Pex5p *in vivo*. (**a**) It is known that the inhibition of the export of Pex5p by the deletion of PEX4 and/or PEX22 induces polyubiquitination and proteasomal degradation of Pex5p. The PEX4/PEX22-deletion induced polyubiquitination of Pex5p can be significantly reduced when the cells express Pex3(N)-Pex4p in combination with Pex22(C)p. (**b**) The Pex3(N)-Pex4p chimera is capable to monoubiquitinate Pex5p *in vivo*. The level of monoubiquitinated Pex5p is reduced in the absence of Pex22p, but is restored in the presence of Pex22(C)p.

We wanted to analyze whether the detected stimulating effect of Pex22(C)p on the E2 activity of Pex4p and the *in vitro* ubiquitination of Pex5p are of relevance *in vivo*. Our data on the specific role of Pex22(C)p in peroxisomal biogenesis and PTS1-protein import so far showed that Pex22(C)p can partially complement the *pex4*Δ deletion strain when Pex4p is anchored by another protein (Fig. 2). It is known that the inhibition of the monoubiquitination of Pex5p caused by the deletion of PEX4 and/or PEX22, or, alternatively, mutation of the AAA-peroxins, induces polyubiquitination and proteasomal degradation of Pex5p [22], [23], [30], [31], [51]. Because of this, the occurrence of polyubiquitinated Pex5p can serve as a biochemical *in vivo* tool to monitor a potential defect in Pex5p export. Here we show that the polyubiquitination of Pex5p seen in *pex4*Δ cells (Fig. 4a, lane 2) can be nearly completely abolished when the mutant cells express Pex3(N)-Pex4p (Fig. 4a, lane 3). When the Pex4p-chimera is expressed in the *pex4*Δ*pex22*Δ strain, the amount of polyubiquitinated Pex5p is only very weakly reduced in comparison to *pex4*Δ*pex22*Δ cells (Fig. 4a, lane 4 and lane 5). However, when in addition to Pex3(N)-Pex4p the Pex22(C)p-fragment is expressed in the *pex4* *pex22* strain, the polyubiquitination of Pex5p is significantly reduced. This indicates that the presence of Pex22(C)p can indeed partially suppress the export defect of Pex5p.

The physiological more important ubiquitination of Pex5p is its monoubiquitination, which represents the canonical signal for export of Pex5p [20]–[22]. In yeast, this modification is catalyzed by Pex4p [22], [23] and we wanted to investigate whether Pex22(C)p also has an effect on this process. To this end, we isolated Pex5p in the presence of NEM in order to inhibit deubiquitination [22], [51]. Moreover we used myc-Pex5p construct, because the N-terminal presence of this tag is known to block polyubiquitination of Pex5p, which would normally occur in *pex4*Δ or *pex22*Δ strains [22]. This enabled us to analyze the effect of the Pex4p- and Pex22p-constructs on the monoubiquitination of the PTS1-receptor. We found that Pex3(N)-Pex4p can monoubiquitinate myc-Pex5p in the *pex4*Δ strain with an efficiency that is comparable to the wild-type situation (Fig. 4b). The level of monoubiquitinated myc-Pex5p is reduced significantly when the Pex4p-chimera is expressed in the *pex4*Δ*pex22*Δ strain. This effect is due to the complete absence of Pex22p, but also indicates that the basic E2-activity of Pex4p does not essentially depend on the presence of Pex22p *per se*. However, when Pex22(C)p was expressed in addition, the level of monoubiquitinated Pex5p increased significantly (Fig. 4b). This demonstrates that Pex22(C)p is required for full activation of Pex4p and efficient monoubiquitination of the PTS1-receptor.

### The stimulating effect of Pex22(C)p on Pex5p monoubiquitination and matrix protein import cannot be enforced or replaced by Pex22(N)p

**Figure 5:**
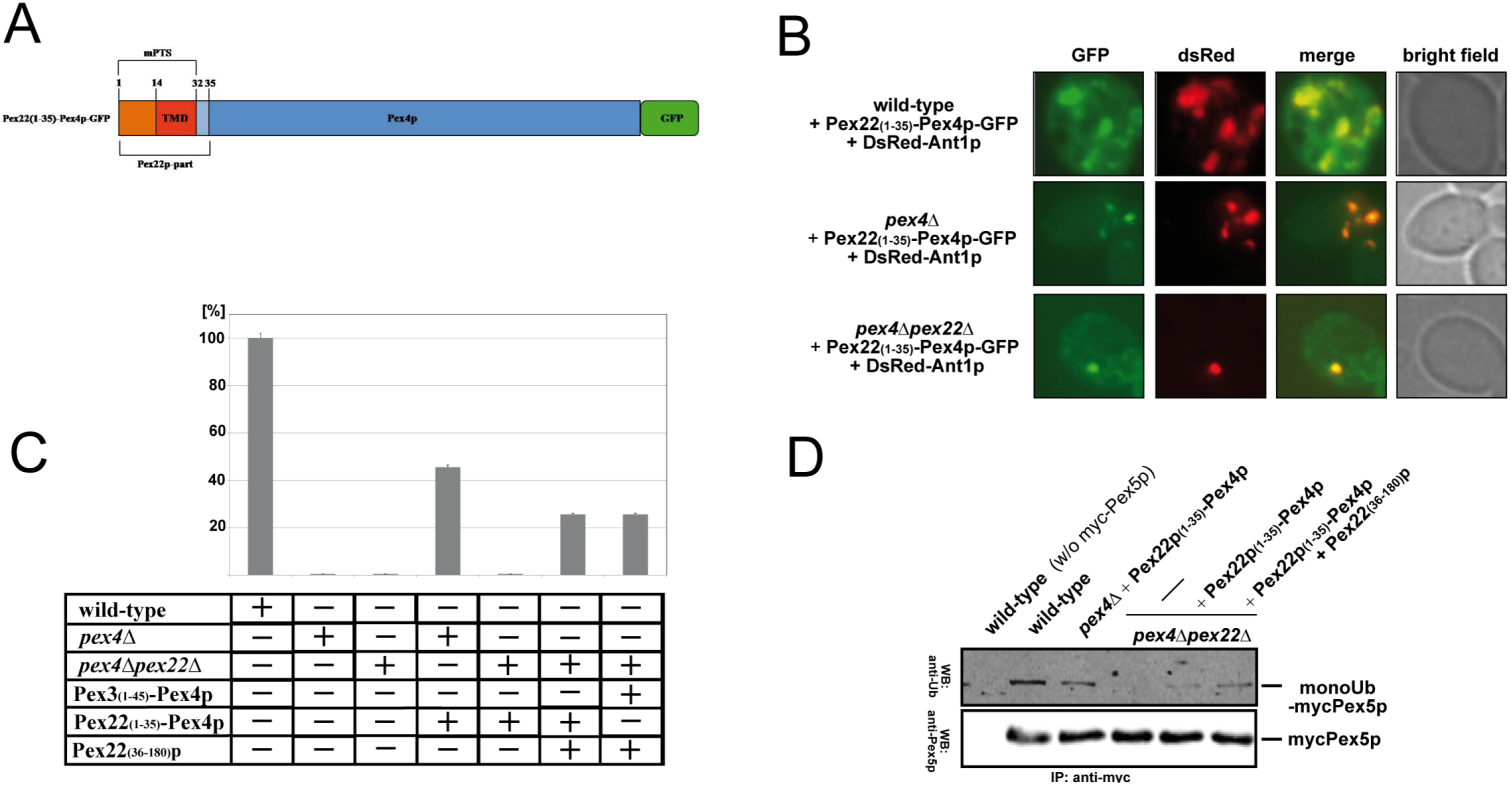
The stimulating effect of Pex22(C)p on Pex5p ubiquitination and peroxisomal function cannot be enforced or replaced by Pex22(N)p. (**a**) Schematic representation of Pex22(N)-Pex4p. The Pex22(aa1–35)-fragment contains the transmembrane domain (TMD) in *red* and the intraperoxisomal part in *orange* fused to full-length Pex4p (*blue*) as well as green fluorescent protein (*green*). (**b**) Pex22(N)-Pex4p-GFP is targeted to peroxisomes. The Pex22(N)-Pex4p-GFP construct was introduced in wild-type, *pex4*Δ and *pex4*Δ*pex22*Δ cells as indicated and co-localization of the chimeric protein with the peroxisomal membrane marker DsRed-Ant1p was analyzed under by fluorescence microscopy. (**c**) The functionality of indicated constructs was tested by analysis of their capability to complement the growth defect of depicted strains. To this end, the Pex3(N)-Pex4p and/or Pex22(C)p or Pex22p encoding information was expressed in the *pex4*Δ*pex22*Δ, *pex4*Δ *or pex22*Δ deletion strains, as indicated. The optical density (OD600) of cells grown in oleate medium was monitored (n = 3 experiments) and the results are presented in % in comparison to the wild-type (+/−standard error of the mean). While Pex22(N)-Pex4p complements the growth defect of the *pex4*Δ strain, it is only capable to regain functionality in the *pex4*Δ*pex22*Δ background, when Pex22(C)p is present. (**d**) The Pex22(N)-Pex4p fusion protein is capable to monoubiquitinate Pex5p *in vivo*. In the absence of full-length Pex22p, the level of monoubiquitinated Pex5p is significantly reduced, which is compensated in the presence of Pex22(C)p.

In the following we tested if also the N-terminal region of Pex22p, thus Pex22(aa1–35)p, in the following designated Pex22(N)p, has an enhancer effect on Pex5p-monoubiquitionation. We generated a fusion of Pex22(aa-1–35) and full length Pex4p (Pex22(1–35)-Pex4p) (Fig. 5a). In a first step, we verified that the chimeric construct is targeted to peroxisomes by fluorescence microscopy. The peroxisomal localization of Pex22(1–35)-Pex4p-GFP is indicated by its congruent fluorescence pattern with the peroxisomal marker DsRed-Ant1p (Fig. 5b). Next, we could show by immunoisolation of Pex4p-complexes and their mass spectrometric analysis that Pex22(N)-Pex4p interacts with Pex22(C)p (data not shown). Furthermore, Pex22(1–35)-Pex4p can complement and thus restore growth ability on oleate medium of the *pex4*Δ strain but not of the *pex4*Δ*pex22*Δ strain (Fig. 5c). However, a partial complementation of the *pex4*Δ*pex22*Δ strain was achieved by combined expression of Pex22(1–35)-Pex4p and the Pex22(C)p fragment (Fig. 5c). These data demonstrate that the presence of the Pex22(1–35)p alone is not enough to compensate the loss of the full length Pex22p, even if it is genetically fused to Pex4p. Only the additional presence of the detached Pex22(C)p enables a partial restoration of peroxisome function.

We next investigated whether it makes a difference for the enhancing effect of Pex22(C)p when Pex4p is fused to the membrane anchor of either Pex3p or Pex22p. The direct comparison of *pex4*Δ*pex22*Δ strains expressing Pex22(C)p and either Pex3(N)-Pex4p or Pex22(N)-Pex4p revealed that both display a similar complementation activity (Fig. 5c). This result demonstrates that Pex22(N)p is not able to replace or enforce the function of Pex22(C)p. This also accounts for the enhancing effect of the C-terminal domain of Pex22p for Pex5p-monoubiquitination as shown in Fig. 5D. The *pex4*Δ*pex22*Δ strain containing Pex22(N)-Pex4p alone displays only a residual level of Pex5p-monoubiquitination, while modification of Pex5p is enhanced, when Pex22(C)p is additionally present.

Taken together, these results indicate that Pex22(N)p, just like Pex3(N)p, functions as the membrane anchor, but is not directly involved in the ubiquitin transfer reaction of Pex4p. The enhancer effect is facilitated only by Pex22(C)p.

## Discussion

In this study, we have analysed the functional interplay of the ubiquitin-conjugating enzyme complex Pex4p/Pex22p and its physiological substrate, the PTS1-receptor Pex5p. We demonstrate that the soluble C-terminal Pex4p-binding domain of Pex22p stimulates the E2-activity of Pex4p both *in vivo* and *in vitro* and thereby enhances the monoubiquitination of Pex5p mediated by the RING-type E3 enzyme Pex12p and supports proper matrix protein import.

The membrane protein Pex22p has been described previously as membrane anchor of Pex4p [32], [33] and it has been shown that the interaction of Pex4p with Pex22p is important for full activity of the E2 enzyme [34], [35]. The crystal structure of Pex4p bound to the soluble part of Pex22p revealed a novel binding interface and block of this interaction resulted in a reduced autoubiquitination of Pex4p *in vitro* and decreased PTS1-receptor ubiquitination *in vivo* and impaired growth on oleate medium [34]. In our study, we analysed the molecular details of the enhancing effect of Pex22p on Pex4p and show that Pex22p stimulates the *in vitro* ubiquitination of its physiological substrate, the PTS1-receptor Pex5p. Thus, the Pex22-induced stimulation of Pex4p is directly transferred to an increased ubiquitination of Pex5p by the RING-ligase Pex12p. This stimulating effect was ascribed to the C-terminal domain of Pex22p. The only other E2 complex that seems to be assembled and regulated in a similar manner is the E2 enzyme Ubc7p and its binding partner Cue1p. The membrane-bound protein Cue1p was first described as anchor and stabilizer of Ubc7p at the endoplasmic reticulum [52]. Moreover, it has been demonstrated that it also functions as activator of the E2 activity of Ubc7p [5], [6]. The truncated version of Cue1p, which contains the binding region to Ubc7p, can enhance the E2 activity by stimulating both the charging of Ubc7p with ubiquitin activated by E1 as well as the transfer of the ubiquitin to the substrate mediated by the interacting E3 ligase Hrd1p [53].

The resemblance of the two ubiquitination cascades is of special interest because there are certain similarities between the ERAD (endoplasmic reticulum associated degradation)-machinery and the peroxisomal exportomer [54]–[56]. However, the available crystal structures indicate that the Pex22p-Pex4p interaction does not display the structural features that resemble the situation of the Cue1p-Ubc7p complex [53]. Therefore, it will be important for further studies to identify a functional interconnection of the Pex22(C)p/Pex4p unit and its assembly with Pex12(RING)p or with the entire trimeric peroxisomal RING-complex (Pex12p, Pex10p, Pex2p) [57].

The dissection of the functional regions of Pex22p revealed that there molecular functions in peroxisome biogenesis are divided among the two domains of the protein. It turned out that the stimulating effect of Pex22p is limited to the C-terminal domain of the protein without contribution of the N-terminal region. The N-terminal region instead comprises the peroxisomal targeting signal and transmembrane domain, required for peroxisomal targeting and membrane-anchoring of the E2-complex. We have used the aa 1–35 fragment of Pex22(N)p for the fusion to Pex4p, while a previous study used Pex22(aa1–54)-Pex4p to complement the *pex4*Δ strain [34]. The complementation seems to be more efficient with the longer construct, possibly indicating a function as hinge-region for the aa 36–54. However, as our Pex22(1–35)-Pex4p is able to complement the biogenesis defect of *pex4*Δ very well, we have possibly identified the minimal region of Pex22(N)p required for its basic function.

Finally, we could show that Pex4p fused to the membrane anchor sequence of Pex22p or Pex3p associates with the soluble C-terminal domain Pex22p at the peroxisomal membrane to a functional assembly in the *pex4*Δ*pex22*Δ background. These switching experiment strengthen the disclosed enhancer function of the C-terminal domain of Pex22p show that the membrane anchor sequence of the assembly is interchangeable. The results merge into a picture of an ubiquitin-conjugating complex at the peroxisomal membrane consisting of three modules, the ubiquitin-conjugating unit, usually contributed by Pex4p, a membrane-anchor domain and an enhancing domain, both usually contributed by Pex22p, with the membrane anchor domain being mutually exchangeable.

The major physiological role of the Pex4p/Pex22p-complex is the monoubiquitination of peroxisomal import receptors. We show that the assembly of Pex4p and Pex22p domains to a functional E2-complex at the peroxisomal membrane results in complementation of the defective matrix protein import and elicits the monoubiquitination of Pex5p in *pex4*Δ*pex22*Δ cells. The monoubiquitination of Pex5p has been described to be an essential prerequisite for the dislocation-step in the receptor recycling pathway and therefore also for the entire matrix protein import process [20]–[22]. It is interesting to note that the Pex4p-chimeras alone still enable a residual modification of Pex5p in the in *pex4*Δ*pex22*Δ strain, but that these cells cannot complement the matrix protein import defect. This complementation is only achieved, when Pex22(C)p is expressed in addition, which results again in an enhancement of the monoubiquitination of Pex5p. These results demonstrate that not only monoubiquitination of Pex5p *per se* is required for peroxisomal matrix protein import, but that also a certain threshold level of modified PTS1-receptor has to be generated by the exportomer. This is in line with the conceptual idea that import and export rates of the receptors have to be balanced because the binding capacities of the peroxisomal membrane complexes seem to be restricted [19], [56], [58]. This connection has been demonstrated for *A. thaliana*, where a strain expressing an functionally impaired PEX6 mutant was only able to facilitate matrix protein import, when this late-acting mutant was combined with an early-acting-mutant, which was a low-expressed allele of the docking protein PEX13 [59]. In line with this, a recently published stochastic computational model of the dynamics of Pex5p ubiquitination and matrix protein translocation in mammals suggests that both processes are cooperatively coupled [60]. Therefore, the residual monoubiquitination detected in our Pex4p-chimeras expressing *pex4*Δ*pex22*Δ strains may most likely drastically reduce the export-rate of the receptors and block the import of new cargo-charged receptors.

This study focuses on the function of Pex22(C)p by utilizing the PTS1-import pathway as experimental system. It will be of interest to elucidate if the described enhancer effect of Pex22(C)p has a similar impact on the ubiquitination of the PTS2-co-receptors and PTS2-protein import.

To conclude, our data demonstrate that the ubiquitination-activity of Pex4p and therefore the Pex12p-dependent monoubiquitination of the PTS1-receptor are stimulated by the C-terminal domain of Pex22p and that this enhancer effect is required to enable the monoubiquitination above a physiological threshold and therefore matrix protein import.

Further experiments will address the functional integration of this Pex22(C)p-mediated enhancer-effect on the translocation of folded proteins over the peroxisomal membrane.
